# Colonization of extended-spectrum β-lactamase-producing Enterobacteriaceae does not affect subsequent infection and liver transplant outcomes: a retrospective observational cohort study

**DOI:** 10.3389/fpubh.2023.1207889

**Published:** 2023-09-19

**Authors:** Chen Shang, Run Yang, Ya Yang, Haomin Zhang, Jianjun Zhang, Qiang Xia, Yuan Gao, Yuxiao Deng

**Affiliations:** ^1^Department of Critical Care Medicine, Renji Hospital, School of Medicine, Shanghai Jiao Tong University, Shanghai, China; ^2^Department of Infection Control, Renji Hospital, School of Medicine, Shanghai Jiao Tong University, Shanghai, China; ^3^Department of Laboratory Medicine, Renji Hospital, School of Medicine, Shanghai Jiao Tong University, Shanghai, China; ^4^Department of Liver Surgery, Renji Hospital, School of Medicine, Shanghai Jiao Tong University, Shanghai, China

**Keywords:** liver transplant, extended-spectrum β-lactamase-producing Enterobacteriaceae, extended-spectrum β-lactamase-producing gram-negative bacilli, colonization, infection

## Abstract

**Objective:**

To investigate the colonization rate of extended-spectrum β-lactamase-producing Enterobacteriaceae (ESBL-E), subsequent infections by ESBL-E and ESBL-producing gram-negative bacilli (ESBL-GNB), and the effect of ESBL-E colonization on clinical outcomes in liver transplantation (LT) recipients.

**Methods:**

This is a retrospective cohort study that included patients who underwent LT at Shanghai Renji Hospital between July 2016 and December 2017. Rectal swabs from LT patients at the postoperative ICU enrollment were screened anonymously for ESBL-E carriage. Demographics data, laboratory indexes, operative complications, and clinical course information were also obtained. The extent of ESBL-E colonization, the subsequent infection rates of ESBL-E and ESBL-GNB, and the clinical outcomes were compared between ESBL-E colonized and non-colonized patients.

**Results:**

In total, 496 liver transplant recipients (387 males) were included in this study. ESBL-E colonization was detected in 240 patients (48.4%). There was no significant difference between the rates of ESBL-E infection (5.8 vs. 3.1%, *p* = 0.143), Ischemia-reperfusion ≥ 3 (27.9 vs. 24.6%, *p* = 0.403), acute kidney injury (39.6 vs. 38.7%, *p* = 0.835), acute rejection (2.1 vs. 1.6%, *p* = 0.664), graft versus host reaction (1.3 vs. 1.2%, *p* = 0.937), duration of hospitalization (22 vs. 23 days, *p* = 0.568), 90-day mortality (7.1 vs. 4.7%, *p* = 0.262) and 1-year mortality (12.9 vs. 9.3%, *p* = 0.265) in patients with and without ESBL-E colonization. Though the ESBL-GNB infection rate was higher in ESBL-E colonized patients (12.1 vs. 6.6%, *p* = 0.037), multivariate analysis showed that ESBL-E colonization did not increase the risk of ESBL-GNB infection (Model 1: aOR 1.755, 95% CI: 0.911–3.380, *p* = 0.093; Model 2: aOR 1.556, 95% CI: 0.761–3.181, *p* = 0.226). The ESBL-producing bacteria spectrum of colonization was significantly different from that of infections occurring after LT, with only three colonization events leading to infection by the same pathogen identified.

**Conclusion:**

ESBL-E colonization in liver transplant patients is not associated with ESBL-E infection, nor is it a risk factor for post-transplant ESBL-GNB infection. Additionally, ESBL-E colonization does not lead to worse prognoses when compared with non-colonized patients.

**Clinical trial registration:**

Chinese Clinical Trial Registry, Identifier [ChiCTR2100043034].

## Introduction

Since the first human orthotopic liver transplantation was carried out in 1963, liver transplantation (LT) has become the most effective choice for the treatment of end-stage liver disease and acute liver failure (1). Enterobacteriaceae that produce extended-spectrum β-lactamases (enzymes that are able to hydrolyze β-lactam antibiotics) have emerged as a significant threat to LT candidates and recipients, as its presence has been linked to increased mortality and morbidity rates (2).

Analysis with regard to extended-spectrum β-lactamase-producing Enterobacteriaceae (ESBL-E) colonization in LT patients has mainly been conducted in Western countries (3), rather than regions such as Southeast Asia, where ESBL-E prevalence is higher (4). Given the significant heterogeneity of ESBL-E reported in different parts of the world and the high volume of transplantation activity globally, the lack of ESBL-E data from regions, such as Southeast Asia, should be underscored (4, 5).

Previous literature has found that almost 40% of ESBL-E colonized LT patients developed an ESBL-E infection during the post-transplant period, compared with 3.5% of non-colonized patients (6), and ESBL-E colonization has been associated with a 12 times greater risk of such infections (7). However, other studies have questioned the benefit of screening for ESBL-E. In Swedish patients with fecal ESBL-E colonization, the risk of ESBL-E bacteremia was estimated to be very low (0.7%) (8), and in general ICU settings, only 10–25% of ESBL-E carriers developed an ESBL-E-related infection (9, 10).

Enterobacteriaceae that produce ESBL are predominantly found in *Escherichia coli (E.coli)* and *Klebsiella pneumoniae (K. pneumoniae)*, though they may also be present in other types of gram-negative bacilli (GNB), including *Pseudomonas* spp., *Burkholderia* spp., and *Acinetobacter* spp. (11–13). The multidrug-resistant nature of ESBL-E may be explained by the production of plasmid-encoded enzymes, which carry multi-resistance genes via plasmids, transposons, and integrons (14). *E. coli* can also transfer plasmids carrying antibiotic resistance genes between co-existing bacteria that are part of the commensal intestinal flora, especially under antibiotic pressure (15, 16); however, these bacteria are not necessarily the same species (14). The extent of the changes in ESBL-carrying bacterial species and the capacity for the possible transfer of ESBL-carrying plasmids in GNB isolates in LT patients remains, to our knowledge, largely unexplored.

In this study, we sought to examine the ESBL-E colonization burden of LT patients, to evaluate the link between ESBL-E colonization and subsequent infections of ESBL-E and ESBL-producing gram-negative bacilli (ESBL-GNB), and to explore the clinical outcomes. To our knowledge, this is the first study to consider ESBL-E epidemiology in LT cases in China.

## Materials and methods

### Study population

This is a retrospective observational cohort study. Patients included in the current study were from the Department of Liver Surgery and Liver Transplantation Center of Renji Hospital, Shanghai Jiao Tong University (Shanghai, China), between July 2016 and December 2017. Patients ≥ 18 years old who had undergone a rectal active surveillance culture (ASC) following a postoperative intensive care unit admission were eligible for inclusion in this study. The exclusion criteria were as follows: death within 72 h of transplantation or retransplant within 90 days of the original operation. If a retransplant was performed at ≥ 90 days after the first LT, the case was included as a separate event. This study was approved by the ethics committee of Renji Hospital, Shanghai, China (Ethics Number: KY2021-019).

### Study design and definitions

Data gathered for this study included patients' baseline anthropometric measurements, laboratory indexes, operative complications, clinical course and survival status via the inpatient and outpatient information collection system.

A rectal swab to screen for ESBL-E was collected anonymously in liver transplant recipients at the time of their postoperative ICU enrollment. ChromID ESBL screening agar plates (bioMérieux, Marcy-l'Etoile, France) and mass spectrometric analysis were used for ESBL-E identification. Post-transplant bacterial infection was defined according to the NHSN/CDC guidelines, factoring in clinical symptoms and culture results as previously described (17). Clinical samples of the cultures were collected when an infection was suspected, and each case was adjudicated independently by two clinicians.

Ceftriaxone lasting for 5 to 7 days was used as standard perioperative antibiotic prophylaxis. In some instances, the surgeon may have modified a patient's prophylactic regimen according to their history of infectious disease. Selective digestive decolonization was not performed. Infection control policy in our center included protective gown and glove usage associated with adequate hand washing, routine screening of ESBL-E, written antibiotic treatment protocol, and continuous surveillance of nosocomial infections.

Three induction immunosuppression (IS) regimens were carried out by our institution, including a standard triple IS regimen, a basiliximab regimen, and a steroid-free regimen. The standard triple IS regimen consisted of a steroid, tacrolimus (TAC), and mycophenolate mofetil (MMF); the basiliximab regimen used basiliximab, steroids, and MMF with delayed introduction of calcineurin inhibitor (CNI); and the steroid-free regimen used basiliximab, TAC, and MMF. The maintenance IS regimen includes combination or separate use of steroids, CNI, sirolimus, and MMF. Following discharge, patients were monitored at the outpatient clinic as previously described (18).

Clinically significant ESBL-E and ESBL-GNB infections at 6 months post-transplant were the primary outcomes identified. Secondary endpoints included Ischemia-reperfusion ≥ 3, acute kidney injury (AKI), hospitalization days, episodes of acute rejection, graft versus host reaction (GvHD), and mortality at 90 days and 1-year post-transplant. Ischemia reperfusion injury (IRI) was defined by measuring aspartate aminotransferase (AST) levels during the first 3 days post-transplant, according to previously published criteria (Group 1: < 600 IU/L; Group 2: 601–2,500 IU/L; Group 3: 2,501–5,000 IU/L) (19).

### Statistical methods

Anthropometric data and laboratory measurements were analyzed using statistical software (SPSS 22.0, SPSS Inc., Chicago, IL, USA). Continuous data were analyzed by *t*-tests or Mann-Whitney U test. Categorical variables were compared using the χ2 test or Fisher's exact test. The 90-day and 1-year survival rates were calculated using the Kaplan-Meier method. Multivariable analysis was carried out using logistic regression. All values are expressed as mean ± standard deviation (SD), median (interquartile range, IQR), number, and percent (%), as appropriate. Two-sided *p*-values < 0.05 were considered statistically significant.

## Result

### Characteristics of the cohort

**Figure 1:**
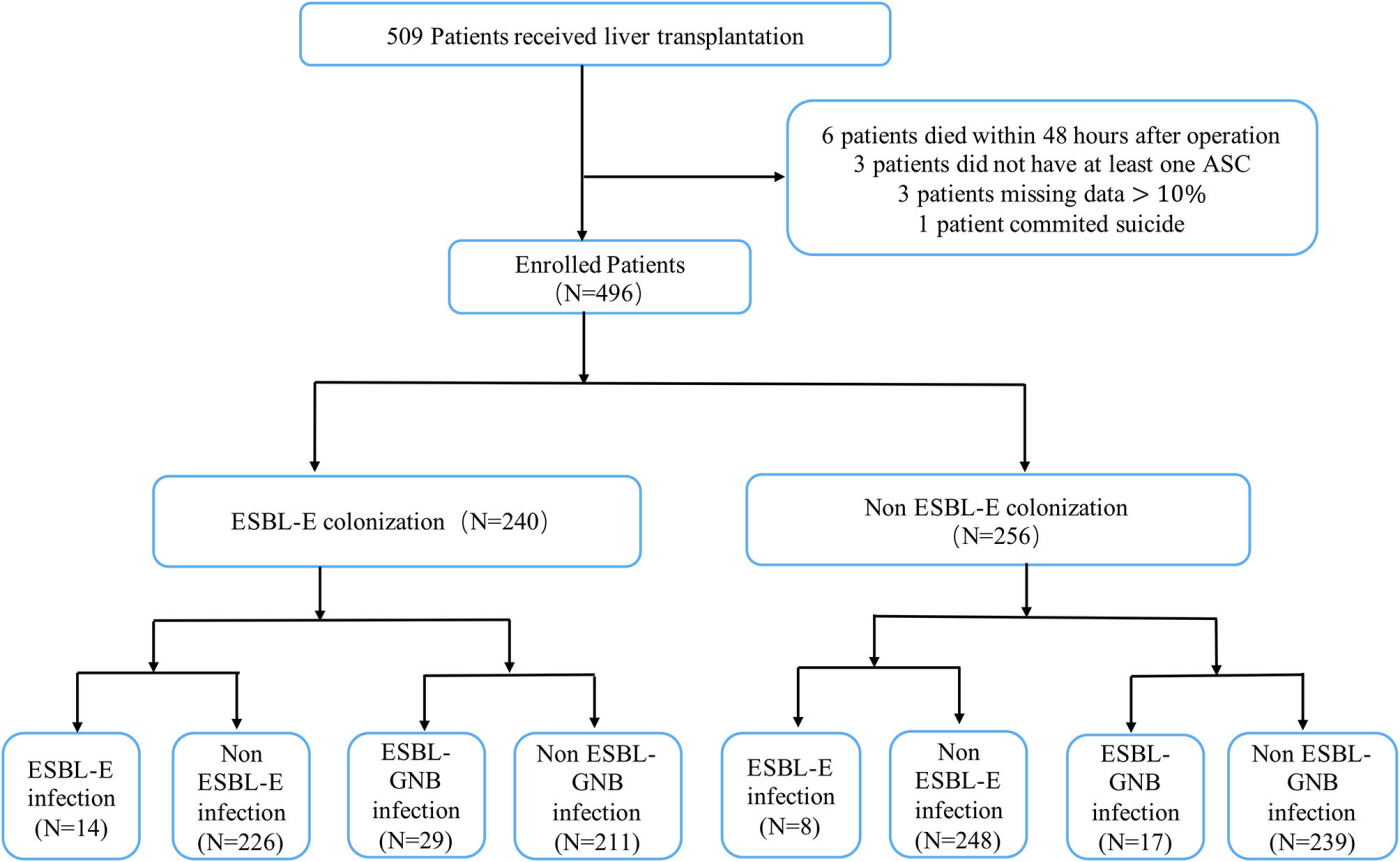
Flowchart of adult patients who underwent liver transplantation in Renji Hospital during the period July 2016 to December 2017.

During the study period, a total of 509 adults who underwent LT at our center during an 18-month period were retrospectively reviewed, of whom six patients died within 48 h after operation, three patients did not have at least one rectal ASC, three patients were missing data >10%, and one patient committed suicide. These 13 patients were excluded from the study. Our final cohort, therefore, included 496 LT recipients with a mean age of 50.1 ± 10.3 years, and 387 (78%) of whom were male. The median MELD score was 13 (IQR 9–20). Prevalence of ESBL-E colonization at the time of transplant was 48.4% (240/496). In the colonized group, 14 patients developed ESBL-E infections and 29 patients developed ESBL-GNB infections, while in the non-colonized group, eight patients developed ESBL-E infections and 17 patients developed ESBL-GNB infections post-transplant. The flow chart of patient selection is shown in Figure 1.

### Anthropometric and perioperative measurements according to ESBL-E colonization

Anthropometric and perioperative measurements of the enrolled patients are summarized in Table 1. The median MELD score was higher in patients colonized with ESBL-E at baseline (14, IQR 10–23) compared with those without colonization (12, IQR 9–18, *p* = 0.027). Anhepatic phase time was longer in ESBL-E colonized patients (40 min, IQR 38–45 vs. 40 min, IQR 35–42, *p* = 0.018). ESBL-E colonization did not vary significantly by age, gender, etiology of liver disease, transplantation reason, previous transplant, PNI, NLR, MLR, or PLR, renal insufficiency, diabetes mellitus, preoperative antibiotic use, pre-transplantation hospitalization days, transfused red blood cells, blood loss, postoperative ICU stay >72 h, postoperative intubation time >72 h, introduction regime, early reoperation rate (All *p* > 0.05).

**Table 1:** Cohort characteristics and demographics.

**Variables**	**ESBL-E colonized (*n* = 240)**	**Non ESBL-E colonized (*n* = 256)**	***p*-value**
**Preoperative data**			
Mean age (SD)	50.1 ± 10.3	51.0 ± 10.4	0.971
Male sex	195 (81.3%)	192 (75.0%)	0.093
MELD score (IQR)	14 (10, 23)	12 (9, 18)	0.027
**MELD score group**			0.006
≤ 15	134 (56.0%)	174 (68.0%)	
16–25	53 (22.0%)	49 (19.1%)	
>25	53 (22.0%)	33 (12.9%)	
**Etiology of liver disease**			0.110
Viral	162 (67.5%)	180 (70.3%)	
Autoimmune	25 (10.4%)	22 (8.6%)	
Alcohol	14 (5.8%)	5 (2.0%)	
Others	39 (16.3%)	49 (19.1%)	
**Reason of transplantation**			0.204
Carcinoma	112 (46.7%)	131 (51.2%)	
Decompensated cirrhosis	90 (37.5%)	98 (38.3%)	
Acute liver failure	38 (15.8%)	27 (10.5%)	
Previous transplant	4 (1.7%)	4 (1.6%)	0.927
PNI (IQR)	40.5 (36.6, 46.9)	40.8 (36.3, 46.5)	0.911
NLR (IQR)	3.21 (1.96, 5.70)	3.02 (1.82, 5.07)	0.270
MLR (IQR)	0.49 (0.33, 0.73)	0.45 (0.30, 0.70)	0.233
PLR (IQR)	88.2 (56.2, 137.2)	88.2 (54.7, 136.7)	0.870
Renal insufficiency	20 (8.3%)	17 (6.6%)	0.473
Diabetes mellitus	44 (18.3%)	52 (20.3%)	0.577
Preoperative antibiotic use	63 (26.3%)	57 (22.3%)	0.300
Pre-transplantation hospitalization, days (IQR)	3 (1, 8)	3 (1, 9)	0.725
**Intraoperative data**			
Anhepatic phase time, min (IQR)	40 (38, 45)	40 (35, 42)	0.018
Red blood cells transfused, units (IQR)	4 (0, 7)	4 (0, 6)	0.673
Blood loss, ml (IQR)	500 (400, 1,000)	500 (400, 800)	0.968
**Postoperative data**			
Postoperative ICU stay > 72 h	10 (4.2%)	5 (2.0%)	0.150
Postoperative intubation time > 72 h	8 (3.3%)	4 (1.6%)	0.200
**Introduction**			0.107
Standard triple induction	15 (6.3%)	29 (11.3%)	
Steroid-free induction	7 (2.9%)	10 (3.9%)	
Basiliximab induction	218 (90.8%)	217 (84.8%)	
Early reoperation	13 (5.4%)	16 (6.3%)	0.693

Data represent no. (%) of patients unless otherwise specified; SD, standard deviation; IQR, interquartile range; MELD, model of end-stage liver disease; PNI, prognostic nutritional index, calculated as [10 × serum albumin (g/dL)] + [0.005 × peripheral lymphocyte count (per mm3)]; NLR, neutrophil-to-lymphocyte ratio; MLR, monocyte-to-lymphocyte ratio; PLR, platelet-to-lymphocyte ratio; Early reoperation, reoperation within 1 month after liver transplantation (20).

### Impact of ESBL-E colonization on predefined endpoints

The primary and secondary endpoints of ESBL-E colonization are summarized in Table 2. ESBL-E colonization was associated with a higher rate of clinically significant ESBL-GNB infection (12.1 vs. 6.6%, *p* = 0.037) but not with ESBL-E infection (5.8 vs. 3.1%, *p* = 0.143). ESBL-E colonization was also not associated with increased risk of Ischemia-reperfusion ≥ 3, acute kidney injury, prolonged hospital-stay, acute rejection, GvHD, 90-day mortality, or 1-year mortality (all *p* > 0.05).

**Table 2:** Cohort clinical outcomes.

**Variables**	**ESBL-E colonized (*n* = 240)**	**Non ESBL-E colonized (*n* = 256)**	***p*-value**
**Ischemia-reperfusion**			
Ischemia-reperfusion ≥ 3	67 (27.9%)	63 (24.6%)	0.403
**Kidney function**			
Acute kidney injury	95 (39.6%)	99 (38.7%)	0.835
**Infections**			
ESBL-E infection	14 (5.8%)	8 (3.1%)	0.143
ESBL-GNB infection	29 (12.1%)	17 (6.6%)	0.037
Hospital stay (IQR)	22 (17, 31)	23 (17, 52)	0.568
Acute rejection	5 (2.1%)	4 (1.6%)	0.664
GvHD	3 (1.3%)	3 (1.2%)	0.937
**Mortality**			
90-day mortality	17 (7.1%)	12 (4.7%)	0.262
1-year mortality	31 (12.9%)	24 (9.3%)	0.265

Data represent no. (%) of patients unless otherwise specified. GvHD, graft versus host reaction.

### Impact of ESBL-E colonization on ESBL-GNB infection

Multivariate analysis aimed at identifying independent variables associated with ESBL-GNB infection at 6 months post-transplant was performed (Table 3). After adjustment for ESBL-E colonization, MELD score, age, diabetes mellitus in model 1, ESBL-E colonization did not increase the risk of ESBL-GBN infection (aOR 1.755, 95% CI: 0.911–3.380; *p* = 0.093). After adjustment for ESBL-E colonization, MELD score, age, diabetes mellitus, pre-transplant antibiotic use, pre-transplant hospitalization days and anhepatic phase time in model 2, ESBL-E colonization still had no significant favorable results for ESBL-GBN infection (aOR 1.556, 95% CI: 0.761–3.181; *p* = 0.226). However, MELD score >25 was associated with an increased risk of ESBL-GNB infection for both models (Model 1: aOR 3.200, 95% CI 1.547–6.619, *p* = 0.002 and Model 2: aOR 3.583, 95% CI 1.568–8.188, *p* = 0.002).

**Table 3:** Multivariate analysis of risk factors for ESBL-GNB infection at 6 months.

**Variable**	**Model 1**		**Model 2**	
	**aOR (95% CI)**	***p***	**aOR (95% CI)**	***p***
**ESBL-E colonization**				
No	1.00 (ref)	/	1.00 (ref)	/
Yes	1.755 (0.911–3.380)	0.093	1.556 (0.761–3.181)	0.226
MELD score		0.006		0.007
≤ 15	1.00 (ref)	/	1.00 (ref)	/
16–25	1.185 (0.502–2.800)	0.698	1.234 (0.466–3.267)	0.672
>25	3.200 (1.547–6.619)	0.002	3.583 (1.568–8.188)	0.002

aOR, adjusted odds ratio; 95% CI, 95% confidence interval; Model 1 was adjusted for ESBL-E colonization, MELD score, Age and Diabetes mellitus; Model 2 was adjusted for ESBL-E colonization, MELD score, Age, Diabetes mellitus, Pre-transplant antibiotic use, Pre-transplant hospitalization days and Anhepatic phase time.

### ESBL-E and ESBL-GNB infections after liver transplantation

During the study period, 44 episodes of ESBL-E infection developed in 22 patients (4.4%) within 6 months following LT (range, 1 to 5 infections per subject). The median time from LT to ESBL-E infection was 16 days (IQR 5–29 days). ESBL-E infection was the first post-transplant infection to occur in 20 out of 22 patients, and the sites of ESBL-E infection were as follows: multi-site (*n* = 11), bloodstream (*n* = 6), intra-abdominal (*n* = 3), and respiratory tract (*n* = 2).

Eighty-eight episodes of ESBL-GNB infection occurred in 46 patients (9.2%) within 6 months following LT (range: 1 to 6 infections per subject). The median time from LT to ESBL-GNB infection was 13 days (IQR 5–23 days). ESBL-GNB infection was the first post-transplant infection to occur in 45 out of 46 patients. The sites of ESBL-GNB infection were as follows: multi-site (*n* = 18), respiratory tract (*n* = 10), bloodstream (*n* = 9), and intraabdominal (*n* = 9).

### ESBL-producing bacterial distribution of colonization and infection

**Figure 2:**
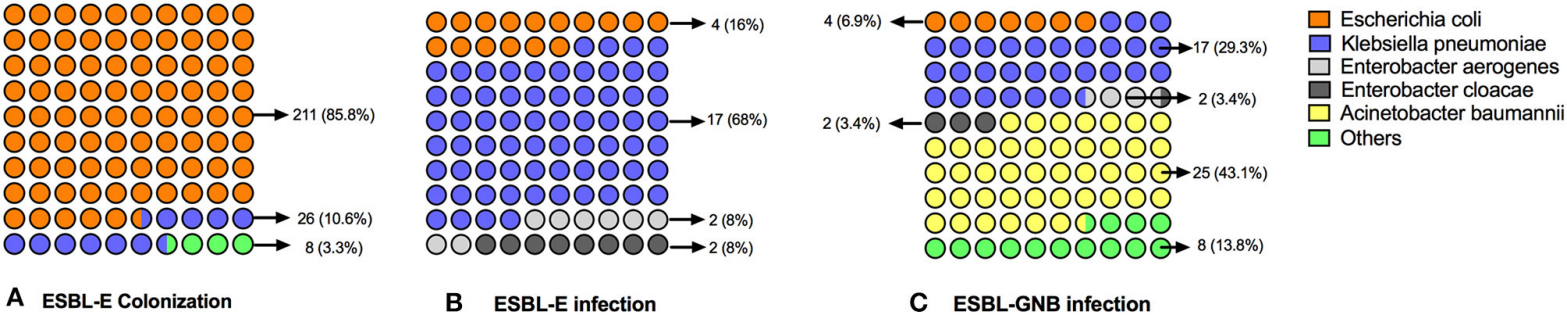
Proportional distribution chart of ESBL-producing bacteria for colonization and infection. A distribution chart showing proportions (with numbers in parentheses) of the predominant ESBL-producing organisms (ESBL-E and ESBL-GNB) annotated by colonization and infection. **(A)** The most predominant ESBL-producing Enterobacteriaceae for colonization was *Escherichia coli* (85.8%); **(B)** the most predominant ESBL-producing Enterobacteriaceae for infection was *Klebsiella pneumoniae* (68.0%); **(C)** the most predominant organism for ESBL-GNB infection were *Acinetobacter baumannii* (43.1%), followed by *Klebsiella pneumoniae* (29.3%).

Of the 240 patients with ESBL-E rectal carriage, 246 strains were isolated; six patients had two distinct ESBL-E isolates. The species distribution of the 246 isolates was as follows: *E. coli* (*n* = 211, 85.8%), *K.pneumoniae* (*n* = 26, 10.6%), *Proteus mirabilis* (*n* = 8, 3.3%), and *Enterobacter aerogenes* (*n* = 1, 0.4%) (Figure 2A).

Of the 22 patients with ESBL-E post-transplant infections, 25 strains were isolated; three patients had two distinct ESBL-E isolates. The species distribution of the 25 pathogen isolates was as follows: *K.pneumoniae* (*n* = 17, 68%), *E. coli* (*n* = 4, 16%), *Enterobacter cloacae* (*n* = 2, 8%), and *Enterobacter aerogenes* (*n* = 2, 8%) (Figure 2B). Three colonized patients developed infections of the same strain (two patients with *E.coli*; one with *K.pneumoniae*).

Of the 46 patients with ESBL-GNB post-transplant infections, 58 strains were isolated; two patients had three distinct ESBL-GNB isolates; eight patients had two distinct ESBL-GNB isolates. The species distribution of the 58 pathogen isolates was as follows: *Acinetobacter baumannii* (*n* = 25, 43.1%), *K.pneumoniae* (*n* = 17, 29.3%), *E. coli* (*n* = 4, 6.9%), *Enterobacter cloacae* (*n* = 2, 3.4%), *Enterobacter aerogenes* (*n* = 2, 3.4%), and other types of GNB (*n* = 8, 13.8%) (Figure 2C).

## Discussion

One important finding from this study was the high prevalence of ESBL-E gut colonization identified at the time of liver transplantation, which accounted for 48.4% of cases in the study, a figure considerably higher than what has been reported in previous literature (13.3~17%) (6, 21). This could also suggest the presence of a particularly heavy ESBL-E burden in our country; as mentioned earlier, the rate of ESBL-E carriers varies greatly across geographic regions. Certain regions in the world, such as Southeast Asia, are known to face a higher ESBL-E burden than others (4, 22). There are relatively few studies investigating ESBL-E colonization in LT recipients and these studies are mainly focusing on Europe (21), and as a result, there is currently no data available that explains the interregional differences affecting the LT population while taking ESBL-E into account. The absence of such research suggests that more comparative studies are needed to assess the ESBL-E colonization rate in endemic areas and in high-risk groups, including LT patients. Our study addresses these gaps in knowledge pertaining to the ESBL-E colonization rate in LT patients in China.

In addition to different epidemiologic features, some aspects of the ESBL-E colonization rate may also be related to the method used to define carriers. For example, in our study, rectal swabs were used to identify carriers, whereas in other studies on LT recipients, positive fecal cultures were used to identify a patient as an ESBL-E carrier (7, 23).

Our data show that patients colonized with ESBL-E had higher median MELD scores at the time of LT (*p* = 0.027). This could be related to the fact that bacteria colonization is more likely to be detected in patients who are sicker; these patients are frequently critically ill, and experience prolonged-hospital stays involving invasive devices and anti-infective agents for both therapeutic and prophylactic purposes, and these factors may also increase the risk of acquiring ESBL-E (7, 24). The median anhepatic phase time was longer in ESBL-E colonized patients (*p* = 0.018). The prolonged anhepatic phase time may also reflect the severity of illness and complexity of the surgery.

Of the 496 transplant patients, 4.4% (22/496) developed ESBL-E infections within 6 months of surgery, an incidence rate comparable with a prior study conducted in one of the largest LT centers in France in which 5.5% of patients developed an ESBL-E infection after LT (7). Our study demonstrates that ESBL-E infections are not often preceded by ESBL-E gastrointestinal (GI) colonization, and the likelihood of ESBL-E carriers developing ESBL-E infections was only 5.8% (14/240) in our LT population, with no significant differences between colonized and non-colonized patients. The infection incidence in colonized patients was lower than that in previous studies, where 44.8 and 39% of patients colonized with ESBL-E developed a subsequent infection (6, 7). Additional risk factor analysis carried out by Logre et al. (6) found that *K. pneumoniae* carriage (compared with other types of ESBL-E) was among the most important predictors for post-LT ESBL-E infection, regardless of the type of infection. In our LT patient cohort, *E. coli* (85.8%) rather than *K. pneumoniae* (10.6%) was the dominant type of colonization, which may partially explain the low rate of subsequent ESBL-E infection. Additionally, many variables can impact the occurrence of infection after LT, especially severity of illness and/or surgery and its complications, and a single rectal swab is unlikely to play a decisive role (25, 26).

To our knowledge, the role of ESBL-E colonization in the development of post-LT ESBL-GNB infections had not been previously demonstrated. This study determined that GI colonization of ESBL-E conferred no increased risk of developing an ESBL-GNB infection after LT; rather, a patient's severity of illness (MELD score > 25) had a greater impact. Although, the impact of MELD score on post-transplant ESBL-GNB infection is revealed for the first time, previous reports indicated that increased pre-transplant MELD score (per 10-point change: HR = 1.59) is a risk factor of developing any infection within 1 year of transplant (27). In prior studies that included both LDLT and DDLT, MELD > 20 was associated with early bacterial infection (28) as well as post-LT septic shock (29).

We herein found that the pathogen spectrum of ESBL-E colonization was significantly different from that of infection after liver transplantation with only three colonization events leading to infection by the same pathogen (all were multiple-site infections). *E.coli* (85.8%, 211/246) was the most prevalent ESBL-producing Enterobacteriaceae for colonization; however, the most common infection causing ESBL-E was *K.pneumoniae* (68.0%, 17/25), rather than *E.coli* (16.0%, 4/25). The most common infection causing ESBL-GNB was *Acinetobacter baumannii* (*A.baumannii*, 43.1%, 25/58), followed by *K.pneumoniae* (29.3%, 17/58). Recent years have witnessed increasing rates of antimicrobial resistance in *K.pneumoniae* and *A.baumannii* in solid organ transplant recipients (30, 31). Our results are consistent with the previous studies wherein *A.baumannii* and *K.pneumonia* were the primary GNB identified in liver transplant recipients (32, 33).

Few prior studies on outcomes of bacterial colonization in liver transplant recipients have been conducted, and these studies reported conflicting results. According to a study conducted in 2008, liver transplant candidates and recipients with VRE colonization had an increased risk of death, whereas those with MRSA colonization had no increased risk of death (34). More recent studies have shown that colonization with MDRO is not associated with increased mortality in short-term follow-ups with LT recipients (35), and the infection free-survival rate following LT does not differ for ESBL-E carriage groups (23). Our results support these more recent findings; that is, colonization of ESBL-E does not impact 90-day or 1-year mortality. This result can be explained by the fact that ESBL-E colonization was not associated with post-LT ESBL-E infection, and neither was it a risk factor for ESBL-GNB infection in our patient cohort.

This study has several limitations. First, the retrospective nature and the single center design was the limitation of the study. Second, the precision of a single rectal swab for detecting colonization may be limited and could have led to the misclassification of colonized patients. However, this would bias the results toward the null hypothesis–that ESBL-E colonization status has a limited impact on the risk of developing subsequent ESBL-producing bacterial infections affecting prognoses. In this article, we found that ESBL-E colonization was not significantly associated with post-LT ESBL-E infection, nor was it a risk factor for ESBL-GNB infection. Additionally, ESBL-E gut colonization did not lead to a worse prognosis compared with non-colonized patients in our study, and the pathogen spectrum of colonization was found to differ significantly from that of bacterial infections. A policy of universal ASC generates both a massive workload for laboratory staffs and substantial expenditures for the hospital system. With the available evidence, we argue against the usefulness of active screening of ESBL-E in liver transplant ICUs where ESBL-E colonization burden is heavy and ESBL- producing *E. coli* predominate. More future researches are needed to verify the clinical value of ESBL-E colonization screening in LT patients.

## Data availability statement

The raw data supporting the conclusions of this article will be made available by the authors, without undue reservation.

## Ethics statement

The studies involving humans were approved by the Ethics Committee of Renji Hospital, Shanghai, China. The studies were conducted in accordance with the local legislation and institutional requirements. The participants provided their written informed consent to participate in this study.

## Author contributions

CS and YD conceived and designed the study. CS and RY wrote the manuscript. YY and HZ collected and analyzed the data. JZ and QX interpreted the results. YG and YD revised the paper. All authors contributed to the article and approved the submitted version.
